# Resin does not safeguard insect DNA during early decay despite preservation of soft tissues

**DOI:** 10.1038/s41598-026-63724-4

**Published:** 2026-09-02

**Authors:** David Peris, Anabela Cardoso, Bastian Mähler, Sabina Karačić, Josefa Cruz, Xavier Franch-Marro, David Martín, Enrique Peñalver, Mónica M. Solórzano-Kraemer, Atahualpa S. Kraemer, Antonio Monleón-Getino, Xavier Delclòs, Ricardo Pérez-de la Fuente, Alba Sánchez-García, Sergio Álvarez-Parra, Jörg U. Hammel, H. Jonas Barthel, Jesús Gómez-Zurita

**Affiliations:** 1 Institut Botànic de Barcelona (IBB), CSIC–CMCNB, 08038 Barcelona, Spain; 2 Section Palaeontology, Bonn Institute of Organismic Biology (BIOB), University of Bonn, 53115 Bonn, Germany; 3 Institute of Medical Microbiology, Immunology and Parasitology, University Hospital Bonn, Venusberg-Campus 1, 53127 Bonn, Germany; 4 Institute of Evolutionary Biology (IBE), CSIC–Universitat Pompeu Fabra, 08003 Barcelona, Spain; 5 Museo Geominero, Instituto Geológico y Minero de España (IGME), CSIC, 46004 Valencia, Spain; 6 Senckenberg Forschungsinstitut und Naturmuseum, Frankfurt, 60325 Frankfurt am Main, Germany; 7 Departamento de Física, Facultad de Ciencias, Universidad Nacional Autónoma de México, Ciudad Universitaria, 04510 Mexico City, Mexico; 8 Department de Genètica, Microbiologia i Estadística, Facultat de Biologia, Universitat de Barcelona (UB), 08028 Barcelona, Spain; 9 Departament de Dinàmica de la Terra i de l’Oceà, Facultat de Ciències de la Terra, Universitat de Barcelona (UB), 08028 Barcelona, Spain; 10 Institut de Recerca de la Biodiversitat (IRBio), Universitat de Barcelona (UB), 08028 Barcelona, Spain; 11 Oxford University Museum of Natural History, Parks Road, Oxford, OX1 3PW UK; 12 Departament de Botànica i Geologia, Universitat de València, 46100 Burjassot, Spain; 13 Institute of Materials Physics, Helmholtz-Zentrum Hereon, 21502 Geesthacht, Germany

**Keywords:** aDNA, Amber, qPCR, Taphonomy, Actualism, Synchrotron imaging, Pine resin, Biological techniques, Ecology, Ecology, Evolution, Zoology

## Abstract

The notion that plant resin acts as an exceptional preservative of insect DNA and soft tissues—functioning as a natural dehydration capsule—has been questioned in recent years, yet empirical support remains limited. We applied an actuotaphonomic approach across a 30-day time series to assess DNA and internal soft tissue preservation in two model species of insects, i.e., *Drosophila melanogaster* (Diptera) and *Tribolium castaneum* (Coleoptera), under two treatments: embedding in fresh pine resin or remaining unembedded and air-dried. Total nucleic acid concentration, relative dsDNA, and quantitative PCR targeting insect and bacterial genes revealed a similar overall DNA decline through time, with no significant differences between treatments, although some resin-embedded replicates degraded faster. Synchrotron tomography showed taxon- and treatment-dependent soft tissue differences. Unembedded specimens of both orders displayed dried internal tissues after 30 days. Resin-embedded fruit flies largely retained intact muscle structure with minimal gas accumulation. Embedded red flour beetles, while maintaining tissue structure, were more poorly preserved, showing diffuse tissue boundaries and abundant gas likely from bacterial metabolism. These findings indicate that soft tissue preservation in pine resin is group-specific, possibly linked to exoskeletal permeability affecting moisture and resin exchange during decay, and suggest that recovering ancient DNA from amber fossil insects is highly improbable.

**Supplementary Information:**

The online version contains supplementary material available at https://doi.org/10.1038/s41598-026-63724-4.

## Introduction

For decades, researchers have dreamed of extracting DNA from fossil organisms. It has been more than 40 years since DNA from an extinct species was successfully recovered for the first time^1^. In that study, the source of biological material was a few-decades-old specimen of extinct quagga zebra. This kickstarted the race to recover increasingly older genetic material and the study of ancient DNA (aDNA), which was significantly accelerated by the use of the polymerase chain reaction (PCR)^2^. The first results from studies involving animals and plants preserved as bioinclusions in amber (fossilized plant resin) suggested that DNA could be preserved in resin for several millions of years e.g., ^3, 4, 5, 6^. DNA preservation in amber was expected based on the assumption that organisms were rapidly embedded by the resin and their soft tissues dehydrated, thus mummified and protected^7,8^. However, these studies faced several criticisms, mainly because of the lack of reproducibility, and their results were interpreted as the product of modern environmental contamination^9,10,11,12^. Thus, the study of aDNA ended up relegated to diverse ancient biological material preserved outside resin, including bones, hair bulbs, skin fragments, fish scales, teeth, or even sedimentary rock samples, usually within the time scale of human evolution e.g., ^11,13,^.

The development of next-generation sequencing (NGS) techniques revolutionized many biological fields, including the study of aDNA^14^. NGS not only facilitates sequencing highly degraded DNA, but also allows for more effective detection of modern contamination^15^. The use of these new techniques prompted renewed attempts to recover aDNA from insects in resin dated between 60 and 10,600 years before present (YBP), but they were unsuccessful^16^. Later, Büsse et al.^17^ reported the successful amplification of aDNA from specimens preserved in a 4,000 YBP Holocene copal (subfossil resin, see^18^). This study provided limited information regarding extraction procedures, contamination controls, and reproducibility, and their positive results should be interpreted with caution.

Peris et al.^19^ extracted and PCR-amplified insect DNA from beetles preserved in two- and six-year-old resin collected in Madagascar from the leguminous species *Hymenaea verrucosa* Gaertn*.*, putting special emphasis on reproducibility and contamination control. Although the results of that work suggested that DNA is preserved within resin samples, there were signs of rapid degradation of organic molecules over a short period. These authors concluded that detection of DNA from organisms preserved in resin is already challenging after just a few years^19^. Using advanced molecular techniques, Modi et al.^20^ subsequently recovered endogenous DNA from one fly preserved in 3510 YBP Colombian copal of *Hymenaea* sp. Their results suggested that, although exceptionally and in very low concentrations, it is still possible to obtain genomic material from resin-embedded organisms^20^. In any case, after more than four decades of research, access to aDNA in any type of resin—be it amber, copal, or Defaunation resin (resin produced after 1760 Common Era sensu^18^)—is still considered a challenge and doubts remain about the actual origin of that DNA. The same controversy does not affect other organic molecules, and there are studies that managed to extract different biomolecules from amber bioinclusions, including carotenoids from Late Cretaceous to Miocene feathers^21^, chitin from an Eocene ant^22^ and a beetle^23^, or amino acids from Late Cretaceous feathers^24^.

Interest in amber bioinclusions is not limited only to the preservation of biomolecules. Organic remains in fossil or subfossil resins usually preserve their three-dimensional shape and, in some cases, even colour (*e.g*.,^25^) and microstructural details (*e.g*.,^26^). However, it is now accepted that although entrapment and fossilization in resin provide substantial mechanical and environmental protection, it does not offer complete sealing^27,28^. Organic remains preserved in resin are affected by diagenetic processes, including groundwater infiltration and internal chemical reactions, which may compromise preservation and promote degradation^29,30^. Furthermore, internal soft tissues in animals typically undergo rapid decay upon death or may be replaced by minerals during fossilization, meaning amber inclusions generally preserve only surface-level anatomy rather than internal organs^28,30^. That being said, it has been shown that in special circumstances amber from diverse localities and ages can preserve the internal, soft tissues of entrapped insects and other arthropods such as digestive tracts, nervous system, muscle fibres, or air sacs with remarkable fidelity for tens of millions of years^8,31,32,33,34^. Consequently, despite the perception of rapid desiccation, antibacterial protection, and exceptional preservation of tissues in amber, the quality of anatomical (and biomolecular) preservation in amber inclusions is highly variable, with processes that control the full preservation spectrum of insects and other arthropods being poorly understood^30,35^.

Taphonomic studies^36,37,38^, including actuotaphonomic approaches using fresh resin^35,39^, have become crucial for understanding preservation and early degradative pathways in resins. The combination of these experimental approaches with advanced analytical techniques, such as Raman spectroscopy or MALDI, and non-destructive imaging techniques —e.g., electron microscopy or microtomographic techniques, including synchrotron imaging^40,41^—have provided a powerful toolkit for multi-scale reconstruction of degradation processes, and they have also advanced our understanding of the physicochemical mechanisms governing biomolecule preservation over geological timescales.

The present study aims to evaluate DNA and internal soft tissue preservation during the first 30 days of entrapment in pine resin of two insect species from disparate orders, true flies and beetles. Two preservation scenarios are considered: specimens decomposing embedded in fresh resin and those unembedded, air-dried. An actuotaphonomic approach combining various experimental techniques —including genomic DNA extraction, quantitative polymerase chain reaction (qPCR) amplification, and synchrotron imaging— is applied.

## Material and methods

### Experimental resin

The resin used to embed the insects was obtained during a resin extraction campaign from March to May 2023 in Vallirana (Barcelona, Spain) using the staking technique on 30 Aleppo pines (*Pinus halepensis* Mill.). Resin extraction was carried out under controlled conditions by Carlos Delgado, the owner of Forest Baix SCCL (Vallirana, Barcelona, Spain), a professional resin extraction company with the correspondent license, using their own pine plantations, who also is the land owner of the plantation, and therefore has permission to obtain resin from his own plantations, and also who identified the pine species from which his company is extracting and commercializing the extracted resin from the identified pines. No acid paste was used during the resin extraction process in order to obtain pure, unadulterated conifer resin. Bark wounds were monitored every two or three days to prevent clogging of the resin canals. Resin was collected in vials and periodically transferred to canning containers. The resin hardened after 15 days, and at the start of the experiment, it had a semi-solid consistency. The hardened resin was fluidified by manually grinding an amount of about 100 g in a mortar.

### Insect samples

A total of 43 specimens of common fruit fly, *Drosophila melanogaster* Meigen (Diptera: Drosophilidae) and 43 specimens of red flour beetle, *Tribolium castaneum* Herbst (Coleoptera: Tenebrionidae) were used. These are two small (2.5–3.5 mm) model insect species, belonging to different orders, and with different exoskeletal properties, *i.e*., soft-bodied and flexible cuticle in the case of *D. melanogaster* versus armoured, highly sclerotized exoskeleton cuticle in *T. castaneum.* Specimens were obtained from strains maintained at the Evolution and Developmental Biology laboratory (IBE, CSIC-UPF, Spain). Only males of *D. melanogaster* were selected to avoid interference from the egg vitellus in female abdomens during the experiment, with sex recognition allowed by strong sexual dimorphism in this species. Individuals of *T. castaneum* were selected regardless of their sex because reliable sex identification in this species is more problematic. Specimens from each insect order were selected randomly for each of the preservation scenarios.

### Sample preparation for DNA and soft tissue analyses

A total of 27 specimens of each insect species were used for the analysis of DNA stability (Fig. S1). All the specimens were simultaneously euthanised by keeping them for 15 min at − 80 °C. Three specimens of each species were used as independent control replicates (Time 0, T0). Each of these T0 specimens was cut longitudinally under aseptic conditions using a sterilized scalpel to expose the internal soft tissues. Dissected specimens were immediately transferred to a sterile 1.5 ml Eppendorf tube and stored at − 80 °C in order to stop biomolecule degradation. The remaining 24 specimens of each species were divided into two experimental sets (12 specimens per species), which were simultaneously subjected to alternative preservation conditions: either embedded in fresh resin or left unembedded, air-dried in sealed tubes. All dead, intact specimens were individually transferred into sterile 1.5 ml Eppendorf tubes and stored closed at 20 °C. Three specimens of each insect species and preservation treatment were successively recovered after one day (T1), three days (T2), seven days (T3) and 30 days (T4) from the start of the preservation treatments and subjected to the same procedure of dissection and preservation at -80 °C storage as described for the control specimens. For specimens embedded in resin, any remainder of resin on their body was manually removed using a sterilized needle prior to dissection. A 180-day time frame had been originally scheduled, but after that long time, the pine resin had become impractical for measuring DNA concentration using the employed analytical techniques due to desiccation and hardening increasing beyond 30 days, and these data were discarded.

The analysis of internal soft tissue preservation was done using synchrotron imaging. A total of 16 specimens for each insect species were used in these experiments. Two sets of eight specimens of each species were also subject to the two preservation scenarios noted above (Fig. S1). At different time intervals prior to synchrotron scanning, *i.e.*, 30 (T4), seven (T3), three (T2) and one day(s) (T1) before scanning, mirroring the absolute times used for DNA analysis, two individuals of each species were euthanised as previously described and either embedded in resin or left to air-dry as noted above in sterile 1.5 ml Eppendorf tubes and stored at 20 °C. The storage countdown began at each independent sacrifice and storage event.

### Total genomic DNA extraction and quantification

Total genomic DNA was extracted from each specimen individually, dissected and preserved as explained above, using the QIAGEN DNeasy Blood and Tissue kit (Qiagen, Hilden, Germany), a spin-column based procedure, following manufacturer’s instructions. The main steps included: overnight incubation of specimens in lysis buffer, followed the next day by buffer conditions adjustment to facilitate selective DNA binding to a silica membrane by centrifugation, membrane washing for elimination of contaminants and enzyme inhibitors and, finally, DNA elution and recovery. All procedures were performed under sterile conditions on a laboratory bench that was cleaned with 10% bleach and 70–80% ethanol and with equipment cleaned with 70–80% ethanol. Eppendorf tubes and filtered pipette tips were sterilized by autoclaving and used for individual samples to prevent cross-contamination among samples. Total nucleic acid concentration was estimated based on absorbance using a NanoDrop 1000 spectrophotometer (Thermo Scientific, Waltham, Massachusetts, USA). NanoDrop measurements may overestimate double-stranded DNA concentration, particularly in the presence of RNA, single-stranded DNA, degraded DNA, or other contaminants, thus providing a maximum boundary for the measure of interest. Nevertheless, despite the fact that Nanodrop measures total nucleic acids, most of the signal in these extractions was likely derived from genomic DNA and, for simplicity, these measurements are referred to as total genomic DNA in the text. The minimum detection limit (LOD) of the NanoDrop ND-1000 spectrophotometer for double-stranded DNA (dsDNA) is 2 ng/μl. Alternatively, genomic DNA integrity and relative dsDNA concentration were assessed with a 4200 TapeStation system (Agilent Technologies, Santa Clara, California, USA), an electrophoresis-based platform using ScreenTape chips. TapeStation measured double-stranded DNA concentration together with fragment size distribution and overall DNA integrity. NanoDrop measurements were used as a proxy for total nucleic acid concentration across all analyses. TapeStation data, which provided variable assessments of DNA integrity due to low initial DNA concentrations below the optimal detection range of the instrument, were used in a complementary and exploratory manner to assess relative trends to those of NanoDrop values.

### Relative measures of insect genomic DNA by qPCR analysis

Relative genomic DNA levels were quantified using quantitative real-time PCR (qPCR) with the iTaq™ Universal SYBR® Green Supermix (Bio-Rad, Hercules, California, USA) in order to investigate the kinetics of specific DNA degradation through time and to compare results between the insects decomposing inside and outside the resin. The analysis was performed in three independent samples for each insect order and time interval using as a template the previous DNA extractions and two different specific primer pairs for small fragments (130–150 bp) of single-copy protein-coding nuclear genes of insect genomes. To ensure consistency across reactions, a master mix was prepared containing the SYBR Green Supermix along with gene-specific forward and reverse primers at a final concentration of 100 nM each. qPCR reactions were performed using 4.4 µL of the genomic DNA extractions per reaction, normalized by volume across all samples. Each sample was analysed in duplicate and pure water was used as a control. Amplification was carried out using an iQ™ Cycler and iQ™ Single-Color Detection System (Bio-Rad), and fluorescence data were collected using the iQ5 Optical System Software v2.0 (Bio-Rad). Relative DNA abundance was determined by comparative quantification, normalized to the amount of genomic DNA present in a reference sample collected at the initial experimental time point (T0). The value at T0 was set to 1, and subsequent time points were expressed as relative abundance to this baseline. Data are presented as relative expression (arbitrary units, a.u.).

In the case of *T. castaneum*, qPCR was performed using primers for the *Rpl32* gene of the homonym ribosomal protein (Tc-Rpl32-F: 5′-CAGGCACCAGTCTGACCGTTATG-3′ and Tc-Rpl32-R: 5′-CATGTGCTTCGTTTTGGCATTGGA-3′;^42^) and the *Egfr* gene, a membrane receptor in the family of tyrosine kinases (Tc-EGFR-F: 5′-TCACGAGCATGTGGTTATGAT-3′ and Tc-EGFR-R: 5′-CTCATTCTCGAGCTGGAAGT-3′;^43^). For *D. melanogaster*, primers targeted *Rpl32* (DmRpl32-F: 5′-CAAGAAGTTCCTGGTGCACAA-3′ and DmRpl32-R: 5′-AAACGCGGTTCTGCATGAG-3′;^44^) and the transcription factor gene *Hr3* (DmHR3-F: 5′-GAGGCTTTTCAATCTGAGCATGAA-3′ and DmHR3-R: 5′-CGATTCCATGTGCAAGATGGAAAT-3′;^45^).

### qPCR analysis of bacterial DNA

Quantification of total genomic DNA extractions showed an apparent increase of the DNA concentration at T4 in some samples of *T. castaneum.* To evaluate the potential contribution of bacterial DNA to the total DNA concentration—perhaps linked to enhanced bacterial activity during tissue degradation—relative bacterial DNA levels were quantified following the same qPCR protocol as before, but using the universal primer combination for the small (135–150 bp) V6-V7 region of the 16S rRNA gene of bacterial genomes^46^. Specifically, the primers used to target this region were: 926F 5′-AAACTCAAAKGAATTGACGG-3′ and 1062R 5′-CTCACRRCACGAGCTGAC-3′^46^.

### Phase contrast synchrotron tomography

For the analysis of internal tissue preservation, 16 specimens of each insect species (with two duplicates per species, time and preservation scenario, Fig. S1) were used. In total, 32 whole-individual scans were performed. Imaging was performed at the Imaging Beamline IBL P05 operated by the Helmholtz-Zentrum Hereon^47,48^, at PETRA III (Deutsches Elektronen Synchrotron, DESY, Hamburg, Germany). For each scan, 1501 projections, equally spaced between 0 and π, were recorded for a tomographic scan using a photon energy of 33 keV. A custom-built 20 MP CMOS detector with an effective pixel size of 0.64265 μm and a sample-to-detector distance of 0.1 m was used to record the images. Tomographic reconstruction has been done by applying a transport of intensity phase retrieval approach and using the filtered back projection algorithm (FBP) implemented in a custom reconstruction pipeline^49^ using Matlab (Math-Works) and the Astra Toolbox^50,51,52^. For the processing, raw projections were binned for further processing two times resulting in an effective pixel size of the reconstructed volume of 1.28 µm. The datasets were processed using the software Irfan View 64 Version 4.53 (Irfan Skiljan, Wiener Neustadt, Austria). Acquired images were studied using Avizo^TM^3D 2024.2 (Thermo Fisher Scientific, Schwerte, Germany) and final high-resolution (300 dpi) figures were produced using Adobe Photoshop CS5 (Adobe Inc., San José, California, USA).

### Mathematical model of total DNA decomposition dynamics

Total and relative genomic insect DNA data through time were fitted to an exponential model (explained in the Supplementary Information) of the form $a{e}^{-bt}$^53^, where *a* and *b ≥ 0* are real parameters and *t* is time. This fit was performed using least squares (using LsqFit.jl), a standard method in these cases^54^, which also yields the overall standard deviation between the data and the resulting curve. In all cases, DNA concentration at time T0 was used as the initial reference time, assuming that genomic material degradation was zero at that time, regardless of preservation treatment, inside or outside the resin. For the relative quantification of genomic DNA obtained through qPCR tests, the genes measured were specific to each insect,therefore, at T0, gene counts were expected to be independent of the preservation treatment. In samples where the total amount of genomic DNA was obtained, the resin itself (or subsequent bacterial growth) could contribute genetic material. Consequently, the total amount of genomic DNA at T0 was considered a lower bound, particularly for the embedded insects; yet, given that the primary source of DNA was the insect tissue samples, the total amount of genomic DNA at T0 should ideally be the same for both embedded and unembedded insects. Furthermore, as the Linear Mixed Model analysis showed (see below), the influence of other genes, such as bacterial genes, was generally small compared to the standard deviation of the model.

Only the temporal changes of insect (or total) DNA levels were assessed with the least squared model and taking into account the previous assumptions, but no mathematical models were applied to the relative measures of bacterial DNA using qPCR, owing to the complexity of the possible models and the insufficiency of data to fit them reasonably. Further details on the possible mathematical models are provided in the Supplementary Information.

### Linear Mixed Model for relative measures of DNA decay

Total genomic DNA measurements yielded data in absolute units, independent of the measured situation. In contrast, DNA measurements obtained by qPCR yielded data in units relative to a reference time, T0. Thus, total genomic DNA could be used to compare the gene count between different measurement groups, while relative genomic DNA could only be used for comparisons between different time points within the same group. This implies that bacterial gene data could not be used directly to determine the relative importance of bacterial DNA compared to the measure of insect DNA. In order to quantify the influence of each DNA component in the samples (target insect DNA, bacterial DNA, and unknown origin), we used a Linear Mixed Model approach^55^. This type of model inherently accounts for relatedness and avoids heteroscedasticity, it provides a robust differentiation between the target degradation dynamics and biological noise. The Linear Mixed Model used was the following:

$$y_{ij} = \beta_{0} + \beta_{1} \cdot(\mathrm{gen-1})_{ij} + \beta_{2} \cdot\left( {\mathrm{gen-2}} \right) + \beta_{3} \cdot(\mathrm{gen-bacteria)}_{ij} + b_{j} + \varepsilon_{ij} ,$$

where ${y}_{ij}$ is the total gene value for observation *i* in sample *j*. gen-1 and gen-2 are the relative measurements of the two genes analysed in *T. castaneum* and *D. melanogaster*, while gen-bacteria is the relative measurement of the V6-V7 rRNA region of bacterial genomes. ${\beta}_{0}, {\beta}_{1}, {\beta}_{2}$ and ${\beta}_{3}$ are the coefficients of the fixed effects (the unmeasured genes and the measured gene groups: gen-1, gen-2, and gen-bacteria). ${b}_{j}\sim \mathcal{N}(0, {\sigma}_{b}^{2})$ is the random intercept associated with sample *j*, modelled as a normal variable with mean 0 and variance ${\sigma}_{b}^{2}$, and ${\varepsilon}_{ij}\sim \mathcal{N}(0, {\sigma }^{2})$ is the residual error, also normally distributed with mean 0 and variance ${\sigma }^{2}$.

If gene groups 1 and 2 accurately represent a subsample of insect total genes, which is expected, since they are single-copy genes, then ${\beta}_{1} + {\beta}_{2}$ should be proportional to the number of insect genome templates for their qPCR amplification, while ${\beta}_{0} + {\beta}_{3}$ should be proportional to the DNA contribution not representing the sampled insect. In particular, ${\beta}_{3}$ should be proportional to the contribution of bacterial DNA in the sample.

All statistical analyses were performed using the Julia programming language^56^. Data manipulation was performed using the CSV.jl and DataFrames.jl packages, linear regressions were performed using LsqFit.jl and MixedModels.jl, and plots were generated using Plots.jl.

## Results

### Genomic DNA concentration

**Fig. 1:**
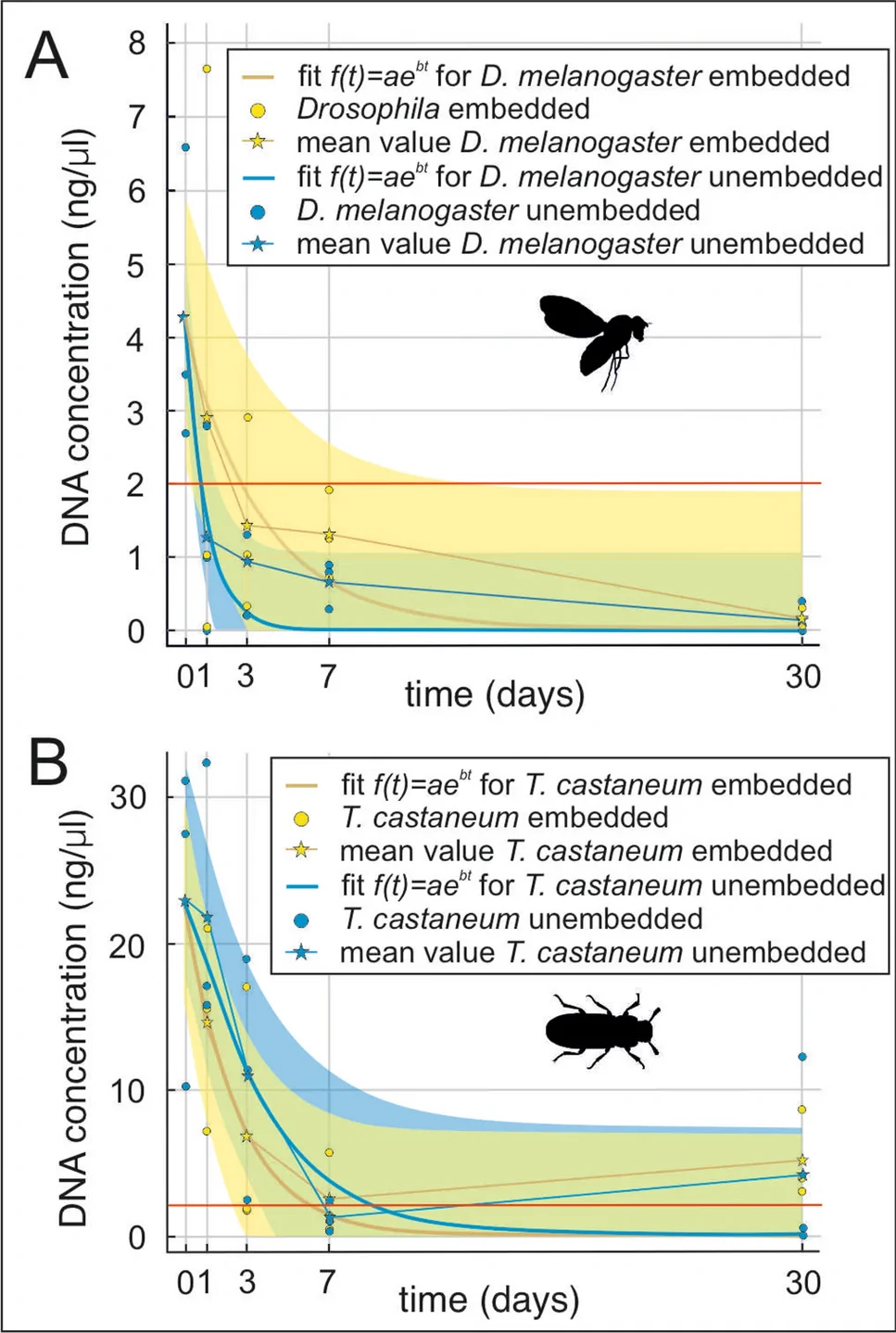
Overall trends of total nucleic acid concentration (ng/µl) over time obtained from independent individual time points based in the NanoDrop quantification. Horizontal red lines at 2 ng/µl represent the lower detection limit for the NanoDrop. (**A**) Total nucleic acid concentration in *Drosophila melanogaster* (Diptera) specimens, both embedded in resin (yellow) and unembedded (blue). Solid lines correspond to the fit of the exponential function, and the shaded areas represent the region around the fitted values within the standard error. (**B**) Total nucleic acid concentration in *Tribolium castaneum* (Coleoptera) specimens, under the same two conditions.

The genomic DNA concentration values obtained for *D. melanogaster* and *T. castaneum* were generally highest at T0 and T1, reaching up to 7.7 ng/µl and 32.3 ng/µl, respectively (Table 1 and Fig. 1). A similar overall trend was observed for both insects and treatments, showing a decrease in DNA concentration over time, with a sharp decline occurring between days 0 and 7, with very few exceptions. At T4, one embedded and one unembedded *T. castaneum* showed higher DNA concentrations than those measured in any specimen at T3. Additionally, some fluctuations were observed among replicates over time and under different preservation treatments, possibly reflecting normal variation of the measuring technique. For *T. castaneum*, DNA concentration was slightly higher in unembedded replicates at T1 and T2 compared to embedded ones, a trend that reversed at T3 and T4. In contrast, for some *D. melanogaster* samples, although DNA concentration remained consistently low, it was slightly higher in embedded samples compared to unembedded ones at T1, T2 and T3 (Table 1 and Fig. 1).

**Table 1:** Total nucleic acid concentration (ng/µl) quantified with NanoDrop for three specimens as independent counterparts at different times (T0, control; T1, one day; T2, three days; T3, seven days; T4, 30 days) from *Drosophila melanogaster* (Diptera) and *Tribolium castaneum* (Coleoptera), under two different preservation scenarios (unembedded or embedded in pine resin).

Species/time	T0 (control)			T1 (1 day)			T2 (3 days)			T3 (7 days)			T4 (30 days)		
*D. melanogaster* unembedded	6.6	3.5	2.7	2.8	1.0 < LOD	0.0 < LOD	1.3 < LOD	0.2 < LOD	1.3 < LOD	0.3 < LOD	0.8 < LOD	0.9 < LOD	0.0 < LOD	0.4 < LOD	0.0 < LOD
*D. melanogaster* embedded				1.0 < LOD	7.7	0.0 < LOD	1.0 < LOD	0.3 < LOD	2.9	0.7 < LOD	1.9 < LOD	1.3 < LOD	0.3 < LOD	0.0 < LOD	0.1 < LOD
*T. castaneum* unembedded	27.4	31.0	10.2	15.8	17.1	32.3	2.5	11.3	18.9	1.0 < LOD	0.3 < LOD	2.4	0.5 < LOD	0.0 < LOD	12.2
*T. castaneum* embedded				15.5	7.2	21.0	1.8 < LOD	1.7 < LOD	17.0	0.5 < LOD	1.4 < LOD	5.7	8.6	4.0	3.1

Only three data points are shown for T0 since at that time point control measurements were taken on specimens prior to starting the preservational treatments (i.e., embedding them in resin or leaving them unembedded). Each measure was obtained from a different individual since the procedure was destructive.

An exponential model fitted reasonably well the observed decrease in DNA concentration in all four experiments, for both insect species and preservation treatments, with a clear overlap of measures within the confidence intervals corresponding to insects embedded in resin and those unembedded (Fig. 1). In other words, the two curves were statistically indistinguishable within the standard error. However, as mentioned above, in the case of *T. castaneum*, the average values showed a small increase at T4 in some values in both preservation treatments. When sample DNA concentrations were derived from TapeStation analyses, all measures were significantly lower and fluctuated in a narrower range of concentrations, making an exponential fit unsuitable for these values (Table S1). However, they showed the same trend of reduction in DNA concentration through time, with low initial values at T0 and T1 and measures below the detection power of the equipment at T3 and T4 (Figs S2, S3).

### Changes in DNA concentration deduced from qPCR analyses

**Fig. 2:**
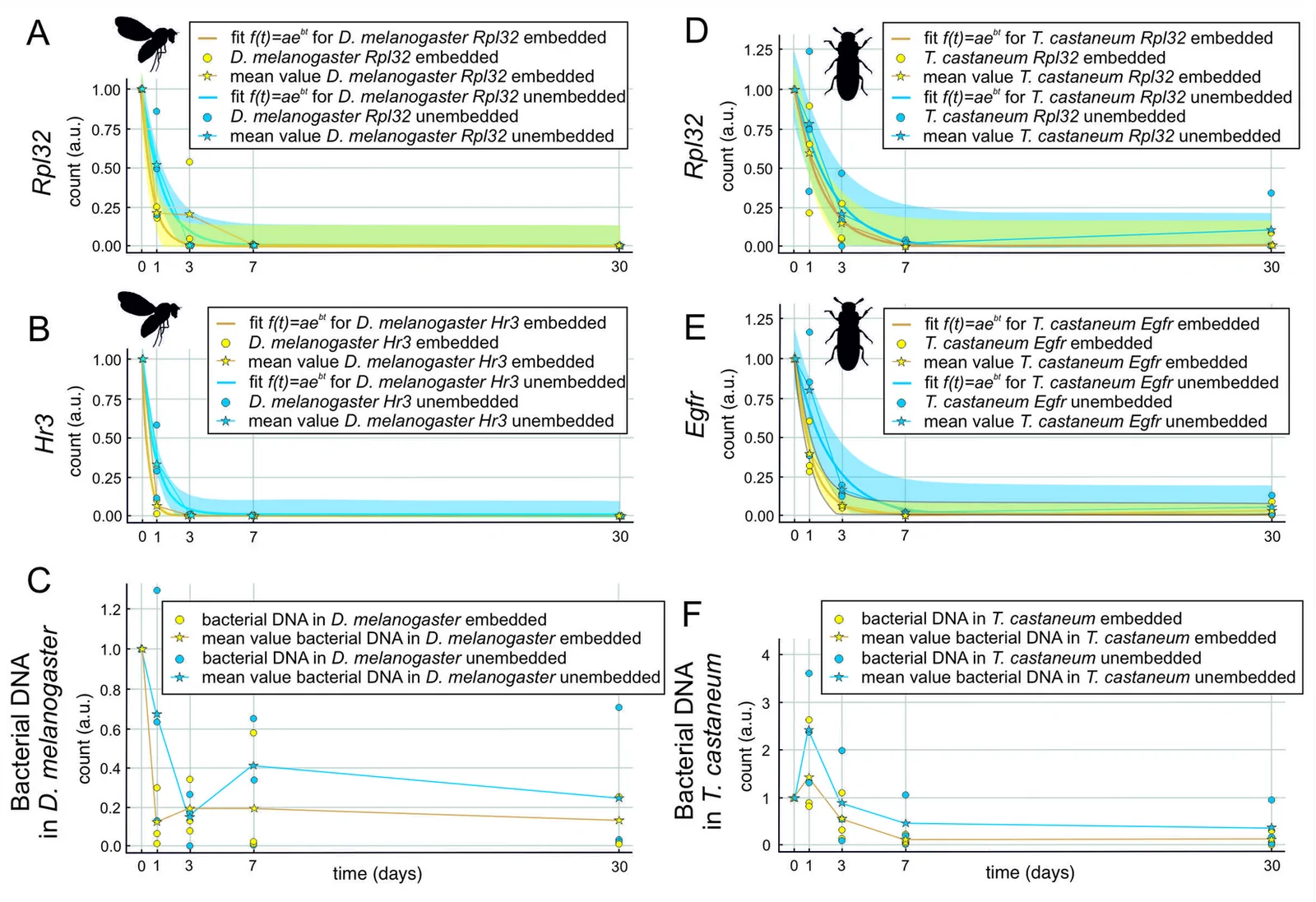
Relative DNA quantification measured in arbitrary units. Data for *Drosophila melanogaster* (Diptera): (**A**) Gen group *Rpl32*; (**B**) Gen group *Hr3*. Data for *Tribolium castaneum* (Coleoptera): (**D**) Gen group *Rpl32*; (**E**) Gen group *Egfr*. Data for bacterial DNA: (**C**) Measured in *D. melanogaster*; (**F**) Measured in *T. castaneum*. Solid curves in A–B and D–E represent the fit of the exponential model, while the mean values for each time point are shown with lines with scatters. Shaded areas represent the region around the fitted values within the standard error.

The inference of relative changes in DNA concentration through time based on the qPCR results consistently showed a decrease over time in all tested scenarios, again representing a good fit to an exponential model of decay. Model parameters and standard deviations are shown in Table 2 and individual qPCR measurements are shown in Table S2. Inferences about genomic DNA degradation were statistically equivalent for gene *Rpl32* in the samples of embedded and unembedded *D. melanogaster* specimens (Fig. 2A), since both curves overlapped within the standard error of one another. However, in the case of *Hr3* data, the curves obtained for embedded and unembedded *D. melanogaster* specimens did not fully overlap, with DNA degradation deduced from lower concentrations appearing to be statistically faster in the embedded flies (Fig. 2B). In the case of *T. castaneum*, inferred results for the kinetics of DNA degradation were statistically equivalent for both genes tested in both embedded and unembedded specimens (Fig. 2D, E).

**Table 2:** Estimated parameters (*a*, *b*) and standard deviation (*σ*) of the fitted exponential function $f(t) = a{e}^{-bt}$ representing DNA concentration decay over time in *Drosophila melanosgaster* (Diptera) and *Tribolium castaneum* (Coleoptera) subject to different preservation treatments measured as total concentration or inferred from specific gene qPCR analyses.

Dataset	*a*	*b*	*σ*
*D. melanogaster* embedded	4.03	0.26	1.89
*D. melanogaster* unembedded	4.19	0.97	1.06
*T. castaneum* embedded	22.50	0.39	6.87
*T. castaneum* unembedded	24.80	0.28	7.41
*D. melanogaster* embedded* Rpl32*	0.99	1.43	0.14
*D. melanogaster* unembedded* Rpl32*	1.02	0.78	0.14
*D. melanogaster* embedded* Hr3*	1.00	2.66	0.18
*D. melanogaster* unembedded* Hr3*	1.00	1.16	0.09
*T. castaneum* embedded* Rpl32*	1.02	0.60	0.16
*T. castaneum* unembedded* Rpl32*	1.05	0.43	0.22
*T. castaneum* embedded *Egfr*	1.00	0.91	0.08
*T. castaneum* unembedded *Egfr*	1.06	0.46	0.19

In the case of bacterial DNA, for *D. melanogaster* samples the tendency was erratic in both embedded and unembedded specimens (Fig. 2C). In this case, the average quantification at T2 in the unembedded specimens was lower than the successive time points (namely T3 and T4). In one of the *D. melanogaster* samples, after 30 days embedded in resin, bacterial DNA was undetectable by qPCR (Table S2). For *T. castaneum* samples, bacterial DNA appeared to decay with time, at least between T1 and T4 (Fig. 2F).

### Linear Mixed Model

Table 3 shows the values of the coefficients ${\beta}_{0}, {\beta}_{1}, {\beta}_{2}$ and ${\beta}_{3}$ for the two preservation treatments of *D. melanogaster* and *T. castaneum*. $({\beta}_{1}+{\beta}_{2}) / \sum {\beta}_{i}$ represents the inferred weight of the pair genes evaluated in each insect species to determine the decay curve, while $({\beta}_{3}) / \sum {\beta}_{i}$ represents the putative weight of bacterial DNA in the DNA pool. $({\beta}_{0}+ {\beta}_{2}) / \sum {\beta}_{i}$ represents the weight of other DNA sources, hypothetically including a fraction representing insect DNA with copy numbers not matching the qPCR results for tested genes. A negative proportion has no biological meaning; thus, these should be considered statistically insignificant. It is noteworthy that the parameter *b* for the two gene types in each insect was, in some cases, similar (for example, for *T. castaneum* unembedded *Rpl32* and *T. castaneum* unembedded *Egfr* it is 0.43 and 0.46 respectively). This implies that the coefficients associated with these genes individually may not correctly represent the proportion of each gene, but in sum may represent the proportion of insect genes. For this reason, it is useful to compare the proportion of bacterial genes with the total proportion of insect genes. In all cases ${\beta}_{3} / \sum {\beta}_{i}$, was either very small compared to $({\beta}_{1}+{\beta}_{2}) / \sum {\beta}_{i}$ (at least one order of magnitude smaller) or negative; that is, the weight of bacterial genes was statistically negligible compared to the insect genes in all cases.

**Table 3:** Parameters of the Linear Mixed Model representing the contribution of different informative sources (βn) for the inference of changes in DNA concentration through time.

	${\beta}_{0}$	${\beta}_{1}$	${\beta}_{2}$	${\beta}_{3}$
*D. melanogaster* embedded	1.22	1.31	2.06	− 0.27
*D. melanogaster* unembedded	0.56	4.04	− 0.38	− 0.13
*T. castaneum* embedded	4.02	13.72	3.58	1.62
*T. castaneum* unembedded	3.16	12.37	5.37	1.65

In the case of ${\beta}_{0}$, the weight of genes was not negligible, but it was comparatively low. The case of *D. melanogaster* embedded in resin is the most striking, as ${\beta}_{0}$ represented just over a quarter of the weight, while the other three cases were closer to an eighth of the weight.

**Fig. 3:**
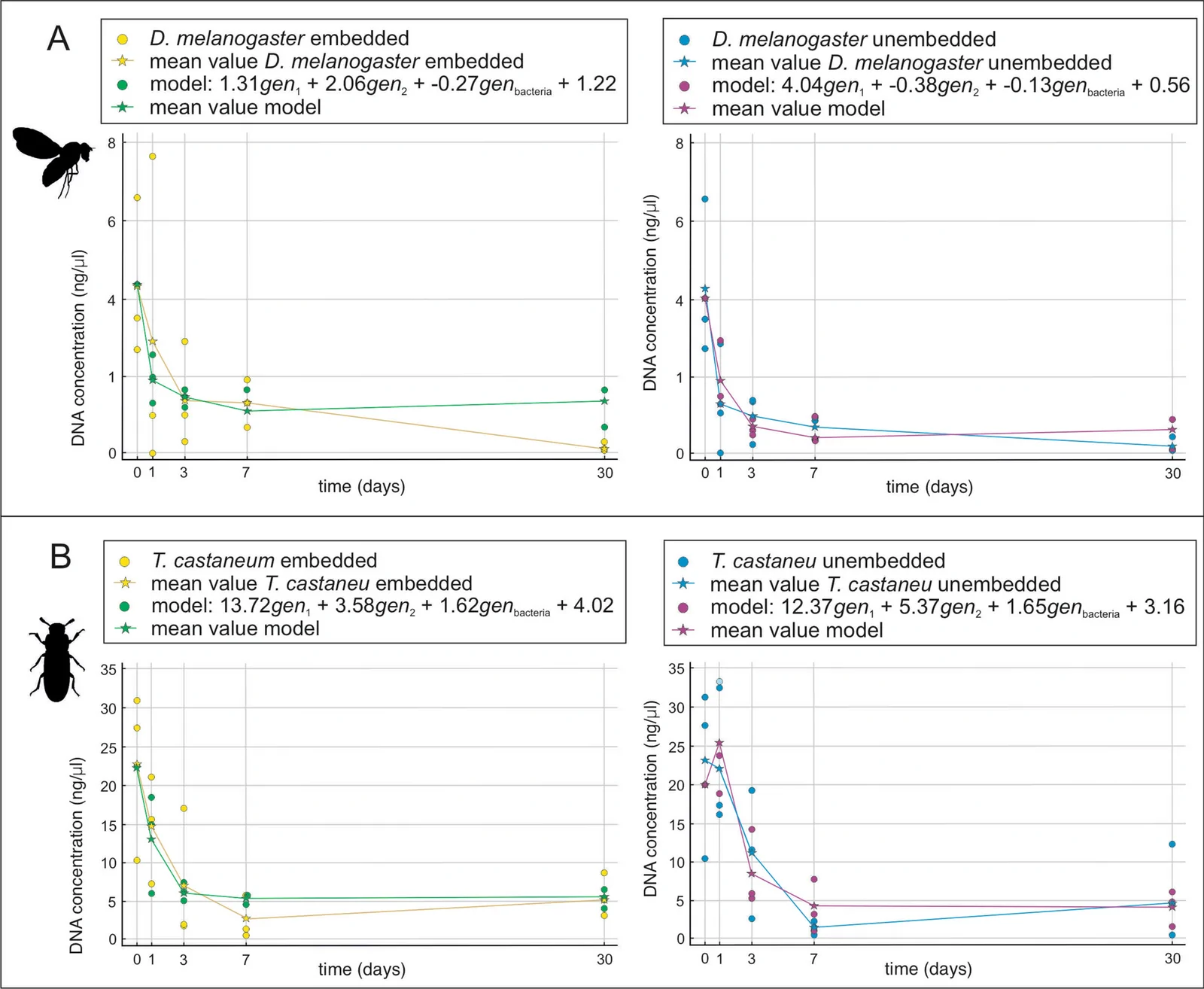
Linear Mixed Model analysis representing the comparison between the genomic DNA obtained after NanoDrop quantification and the mixed model. (**A**) *Drosophila melanogaster* (Diptera) embedded and unembedded. (**B**) *Tribolium castaneum* (Coleoptera) embedded and unembedded.

Figure 3 shows inferred changes of DNA concentration through time for *D. melanogaster* (Fig. 3A) and *T. castaneum* (Fig. 3B) in both embedded and unembedded specimens, relative to the obtained values fitted to their respective Linear Mixed Models. Remarkably, the model was accurate despite being based on only three genetic groups, one of which (bacteria) being negligible.

### Phase contrast synchrotron tomography

**Fig. 4:**
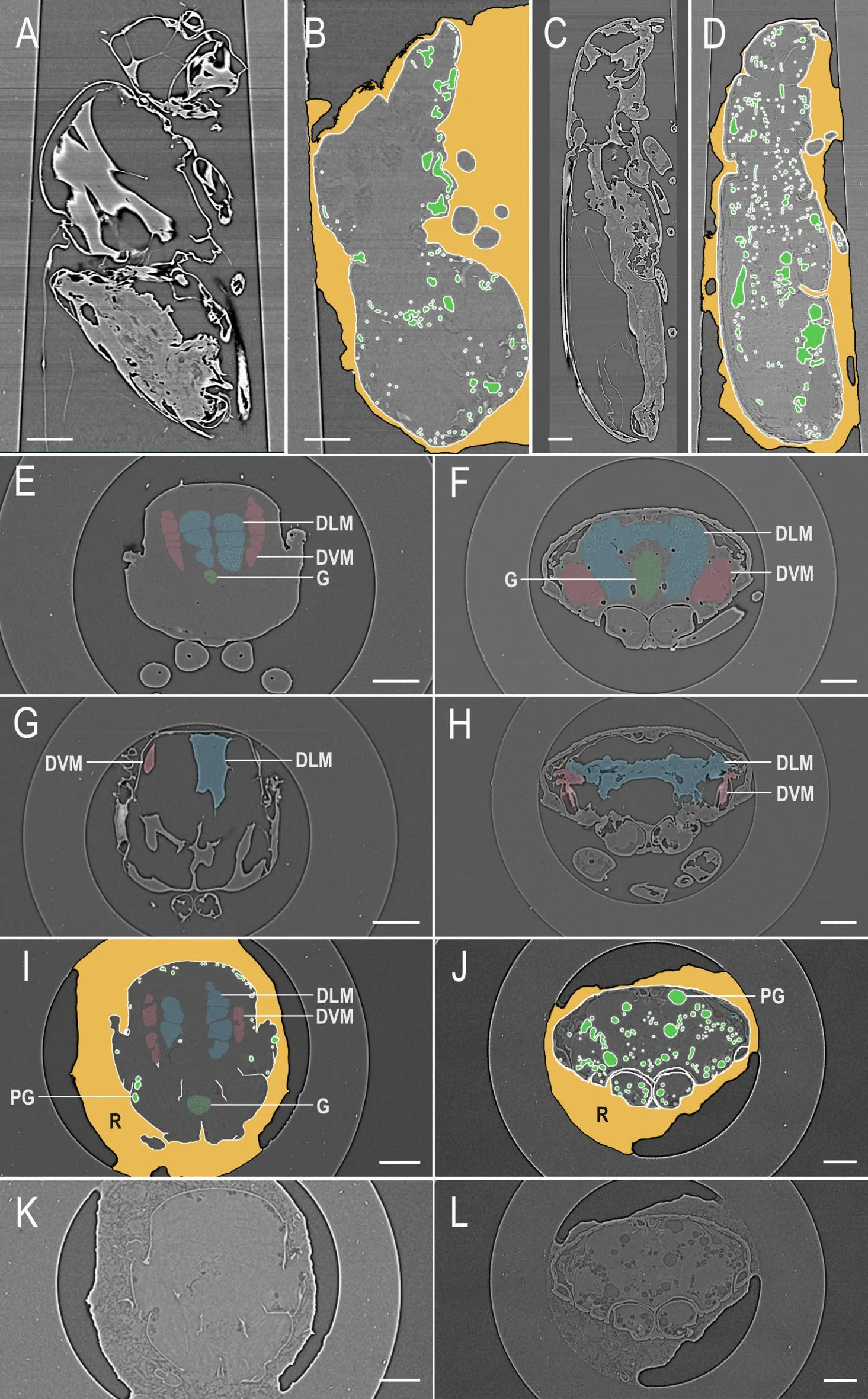
Sagittal (**A**–**D**) and thoracic (**E**–**L**) cross section synchrotron images of *Drosophila melanogaster* (Diptera) (**A**, **B**, **E**, **G**, **I**, **K**) and *Tribolium castaneum* (Coleoptera) (C, D, F, H, J, L). (**A**,** G**). Unembedded *D. melanogaster* at day 30 of the experiment (T4). (**B**, **I**,** K**). Embedded (in pine resin) *D. melanogaster* at T4. I and K are the same image with and without editing, respectively. (**C**,** H**). Unembedded *T. castaneum* at T4. (**D**,** J**, **L**). Embedded *T. castaneum* at T4. J and L are the same image with and without editing, respectively. (**E**) Unembedded *D. melanogaster* at day 1 of experiment (T1). (**F**) Unembedded *T. castaneum* at T1. Abbreviations: DLM, dorsolateral muscles; DVM, dorsoventral muscles; G gut; PG, putrefaction gas observed in the specimens embedded in resin (coloured in green); R, resin (coloured in yellow). All scale bars are 0.1 mm.

At day one (T1), *D. melanogaster* and *T. castaneum* air-dried specimens maintained internal soft tissue integrity, without signs of gas bubbles (Fig. 4E, F). On the other hand, resin-embedded specimens started to accumulate gas bubbles at this point in time, with gas volume, established as both the number and the size of bubbles, generally increasing over time and peaking after 30 days of treatment (T4) (Figs. 4B, D, I, J). At T4, unembedded specimens of both species showed good preservation of their exoskeleton cuticle, but also a noticeable shrivelling of internal soft tissues across all body regions (Fig. 4A, C, G, H). Remarkably, dorsolateral and dorsoventral muscles could still be identified in both *D. melanogaster* and *T. castaneum*, although they appeared contracted and desiccated (Fig. 4G, H). In comparison, resin-embedded *D. melanogaster* specimens at T4 showed only partial preservation of exoskeleton cuticle (Fig. 4B), but good preservation of internal soft tissues, with muscles remaining visible and retaining their original morphology (Fig. 4I, K). Conversely, embedded *T. castaneum* specimens at T4 showed complete preservation of exoskeleton cuticle (Fig. 4D), but a worse preservation of the internal soft tissues based on their less-defined boundaries, with a greater accumulation of gas bubbles (Figs. 4J, L).

## Discussion

Taphonomic research is generally considered to be a long-term endeavour because of the time scale at which factors affecting preservation typically manifest their effects^57^. However, our experimental approach revealed a significant impact on the preservation of DNA and soft tissues after only 30 days or less, consistent with previous findings^35^.

In the case of DNA, the degradation of this biomolecule followed a similar trajectory in both resin-embedded and unembedded insect specimens as measured with different strategies. Overall, no significant differences demonstrate that pine resin does not effectively preserve insect DNA during early decay compared with samples unembedded, air-dried. In fact, a weak tendency is observed in the opposite direction, with the degradation of DNA being faster in insects embedded in resin than those kept air-dried based on results from one of the qPCR analyses and their mathematical fit. Even though NanoDrop DNA quantification is only indicative (i.e., measurements cannot be used as absolute values), the obtained replicated measures are nevertheless useful to investigate the trends of interest. Attempts to obtain more accurate quantification were carried out using TapeStation measurements (Table S1), and although these values should also be interpreted with caution because some DNA concentrations were below the optimal detection range of the instrument, the results revealed a similar degradation trajectory and no significant differences between resin-embedded and unembedded specimens across all approaches (Fig. S2). When the absolute framework for DNA concentration is provided by these alternative measures, the Linear Mixed Model for relative contributions of DNA sources also fits the data (Fig. S3), although with different weights attached to each group (Table 3; Fig. 3).

Despite the general decay pattern of DNA concentration, two *T. castaneum* specimens (one from each preservation treatment) exhibited an anomalously high DNA concentration at time point T4 relative to T3 (Table 1, Fig. 1B). Combined with the observation of significant gas accumulation in resin-embedded *T. castaneum* samples in this same time framework, we considered whether the apparent increase in DNA concentration could be the result of a sudden proliferation of metabolically active bacterial populations. However, qPCR analyses did not reveal a significant increase in bacteria DNA through time (Fig. 2F), and the apparently anomalous observations in *T. castaneum* might be the result of standard deviation in DNA measurements. A similar interpretation can be made for the measured fluctuations of bacterial DNA in the case of *D. melanogaster* samples (Fig. 2C). The fast DNA decline inferred from the analysis of the *Hr3* gene in *D. melanogaster*, significantly faster in resin-embedded specimens (Fig. 2B), is also of interest. The same result is also reflected by the marginal contribution of *Hr3* data to the overall trend measured in the Linear Mixed Model analysis (Fig. 3A). Because there is no particular DNA degradation process that may affect genes differently, these different behaviours may be attributed to qPCR differential performance.

Overall, the Linear Mixed Models show that in all the experiments, measured DNA primarily belongs to the insects, with about one-eighth due to bacteria (Fig. 3, Table 3), supporting the general scenario of fast exponential decline of insect DNA quality and quantity through time, with a major breakdown in the first three days upon insect death. This process occurs regardless of the preservation medium at similar rates at best, but with indications that it may be faster in resin-entombed individuals.

Previous experiments suggest that, although DNA retrieved from resin-embedded insects is highly fragmented and present in very low concentrations, it is still possible to obtain genomic material from specimens up to 3,500 years before present (YBP)^20,19^. These findings do not contradict the results of the present study. Here, we conclude that DNA decay does not differ between resin-embedded organisms and unembedded ones. Although this effect was observed after only three days of experimentation, we did not assess the upper time limit for DNA preservation, which may extend at least to several thousand years^20^. As observed, a range of biological and environmental factors, both intrinsic and extrinsic to the specimens, may influence these results. But critically, our results suggest that the resin embedded/unembedded condition is not one of them.

In air-dried specimens of *D. melanogaster* and *T. castaneum*, all internal soft tissues dehydrated and contracted in less than one month due to fluid exchange with the external environment (Fig. 4G, H). Such desiccation most likely took place through the spiracles, which connect to the tracheal system permeating the body^58^, as well as through the gut openings once inner moisture reached the digestive lumen during decomposition. However, immersion in fresh resin occludes the respiratory system and can be generally considered to create either a barrier to diffusion of fluids outside the body of the embedded organisms or to create new gradients, stronger or weaker depending on the impermeability of the cuticle exoskeleton^35^. Previous studies on contact insecticides^59^ and fossil insects preserved in Zhangpu amber^60^ indicate that the properties of the exoskeleton in combination with the structure of the tracheal system have an impact on the preservation of the soft internal tissues. However, the complex interactions of fluid or gaseous compounds with the tissue of the embedded organisms remains to be assessed. Adult beetles typically possess a robust exoskeleton, which may explain their greater structural integrity in the amber record compared to insects with softer exoskeleton, such as adult flies. Yet, the cuticle not only provides structural support, but it exhibits different degrees of permeability to water and gases to and from their surroundings^61^. Through the scans, we observed that the fluid resin did not penetrate the interior of any of the studied specimens. Moreover, it is possible that the highly sclerotized exoskeleton cuticle of *T. castaneum* creates a more sealed and insulated environment able to retain higher body moisture. Soft tissue decay through microbial metabolism inside of a highly sealed organism could explain the proliferation of metabolic gas bubbles and a higher decomposition rate of internal tissues as observed in these beetles (Fig. 4). Conversely, the experiments also showed that the thinner exoskeleton cuticle of *D. melanogaster* is only partially preserved after several weeks in resin. This structural fragility may allow chemical interactions between the resin and the internal organs of these flies as well as molecular diffusion in and out of their bodies, effectively curbing bacterial activity and decay processes. This would explain why, after 30 days embedded in resin, the internal soft tissues of the *D. melanogaster* specimens exhibited good preservation, retaining a morphology similar to freshly euthanised individuals (Fig. 4E, I). Complementary experiments made by one of us (B. Mähler) on moths and butterflies support that internal soft tissues are preserved after resin embedding in arthropods with a relatively low degree of cuticular sclerotization. Since our findings indicate that inclusion in fresh pine resin can safeguard the internal soft tissues of small insects with a delicate exoskeleton cuticle such as fruit flies against dehydration and decomposition, at least during the first weeks of entombment, the prevailing hypothesis that resin acts as a dehydrating capsule, fixing biomolecules and tissues upon entombment^8,62^, should be re-evaluated.

Previous experiments have shown that embedding insects in resins of different types and characteristics may influence decay^30,35^. It is noteworthy that pine resin—specifically *Pinus sylvestris* L. resin—was found to be ineffective at preventing the decay of internal soft tissues in fruit flies after 18 months of entombment^35^. Although these results differ from ours, perhaps a longer experimental time would have led us to similar conclusions. It is also possible that, following McCoy et al.^30,35^, differences in the physical and chemical characteristics, even between *P. sylvestris* and *P. halepensis* resins, could impact preservation. Volatile resin compounds may play an important role during the early phases after entrapment. In that regard, using resin from a single species as well as the timespan between resin collection and experimental start may have limited the scope of our results. The concentrations of phenolic compounds in resin can vary depending on tree species, season, and environmental conditions^63^. In addition, the antimicrobial properties of the resin could also play a role^64^. Therefore, our results are difficult to be generally extrapolated without additional and independent confirmation. Future research may benefit from comparative experiments using a wider range of resins from both gymnosperms and angiosperms to assess potential differences and effects in bioinclusion preservation. Additionally, the effect of body size was not investigated. The selection of two small insect species, *D. melanogaster* (Diptera) and *T. castaneum* (Coleoptera), might have also constrained the results, as larger organisms may experience different decay dynamics, like moisturization rather than dehydration. Also, the small size of these species compromised the ability to accurately assess DNA integrity from single individuals, and pooling of multiple individuals for DNA extraction should increase DNA yield and improved results. Testing for other variables in future analyses, such as environmental temperature or resin exposure to sunlight, will provide a more comprehensive view on the processes impacting preservation in resin.

## Conclusions

We provide evidence that the entombment of insects in pine resin does not prevent the early degradation of DNA, even when the integrity and preservation of the internal soft tissues may remain intact. In fact, our data appear to lean towards resin actively promoting molecular decay rather than providing a protective microenvironment in the early stages of decay.

Inclusion in fresh pine resin can, at least during the initial weeks, safeguard the internal soft tissues of small insects with a delicate exoskeleton cuticle, such as fruit flies, against dehydration and decomposition. In insects with a more robust exoskeleton cuticle such as red flour beetles, internal soft tissues showed a higher degree of decay based on their appearance and the higher accumulation of gas bubbles attributed to decomposition. The differential preservation of internal soft tissues between these two insect groups suggests that soft tissue preservation is contingent not only on external environmental conditions, such as moisture, temperature, or the chemical composition of resin, but also to some degree on the anatomical and physiological characteristics of the embedded organism.

While significant efforts were invested in searching for DNA or other organic molecules preserved in fossil or subfossil resins in the past, experimental studies on the processes behind the preservation of biomolecules inside fresh resin has been limited despite their potential to shed light on the processes of preservation in the amber record. Several challenges of these experimental studies could be addressed in future studies by: (1) increasing sample replication, (2) testing across different resin types, (3) incorporating additional experimental variables, such as temperature and controlled aging factors, and (4) incorporating a broader taxonomic sampling. In any case, our results overall support with experimental taphonomic data that organic preservation in the amber record may not be as exceptional as classically regarded. For now, and as Grimaldi^65^ stated: “*Jurassic Park will always remain fiction*.”

## Electronic Supplementary Material

Below is the link to the electronic supplementary material.

## Data Availability

All the data needed to evaluate the conclusions of the paper are available in the paper and in the supplementary information files.
