# Supplementary material for: Resin does not safeguard insect DNA during early decay despite preservation of soft tissues
Source: Sci Rep. 2026 Sep 2;16:24920. doi: 10.1038/s41598-026-63724-4 (PMC13538600; doi:10.1038/s41598-026-63724-4)
Supplement: Supplementary file 1 [file 41598_2026_63724_MOESM1_ESM.pdf]

SUPPLEMENTARY INFORMATION

Resin does not safeguard insect DNA during early decay despite preservation of soft tissues

David Peris, Anabela Cardoso, Bastian Mähler, Sabina Karačić, Josefa Cruz, Xavier Franch-Marro, David Martín, Enrique Peñalver, Mónica M. Solórzano-Kraemer, Atahualpa S. Kraemer, Antonio Monleón-Getino, Xavier Delclòs, Ricardo Pérez-de la Fuente, Alba Sánchez-García, Sergio Álvarez-Parra, Jörg U. Hammel, H. Jonas Barthel, Jesús Gómez-Zurita

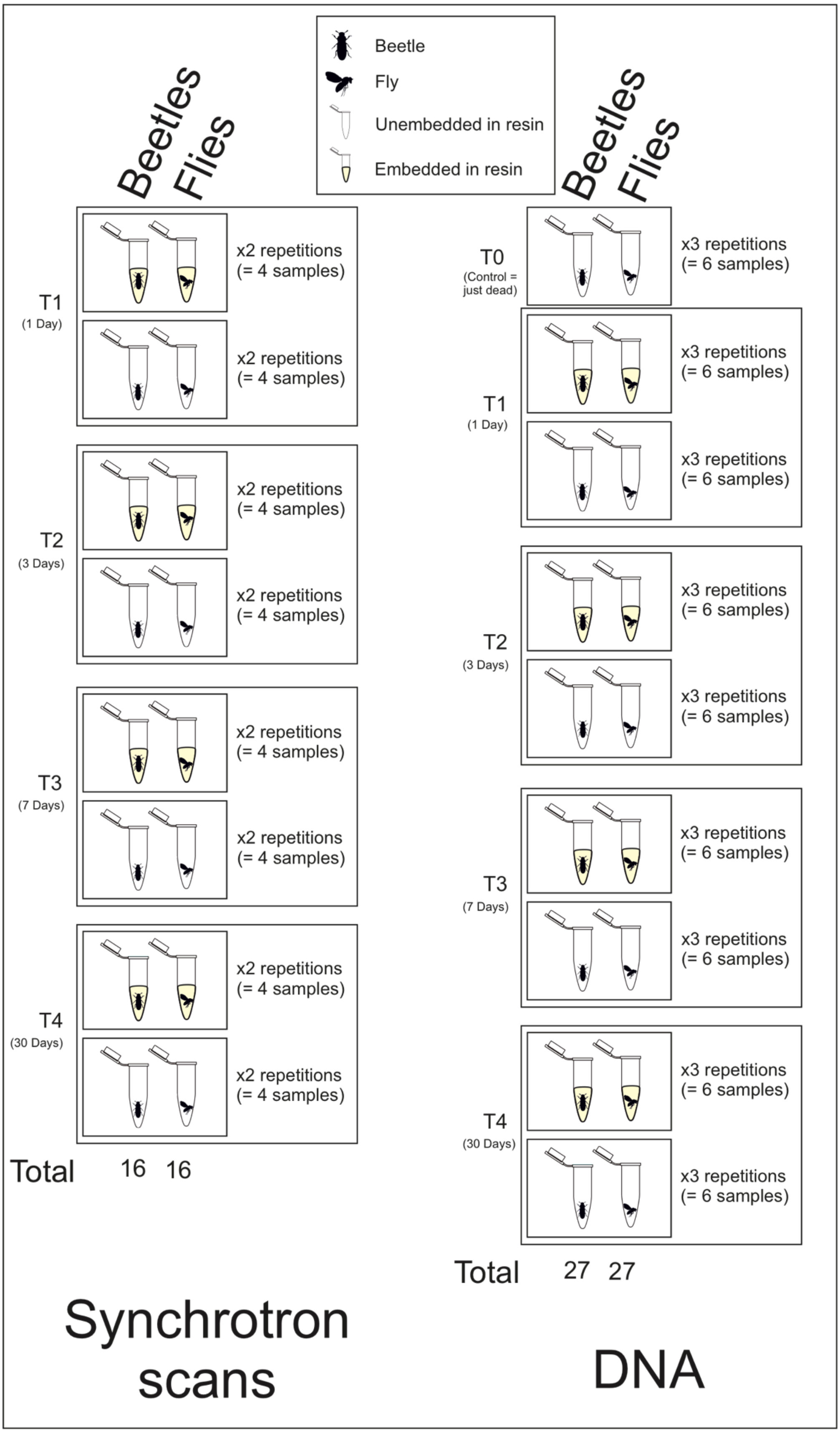

Figure S1. Schematic illustration of the samples and repetitions used for each time point and preservation scenarios for both DNA and soft tissue (synchrotron) analyses.

**Species/Time**	**T0 (control)**			**T1 (1 day)**			**T2 (3 days)**			**T3 (7 days)**			**T4 (30 days)**		
***D. melanogaster* unembedded**	1.75	1.16	1.27	1.33	1.06	2.44	1.43	0.684	1.2	1.13	1.23	1.24	0.828	0.926	1.37
***D. melanogaster* embedded**				1.45	1.42	1.11	1.15	0.908	1.94	1.13	0.755	1.12	0.952	1.07	1.32
***T. castaneum* unembedded**	4.13	3.57	1.32	3.01	1.73	11.8	1.76	1.93	3.14	1.76	1.44	2.4	1.43	1.38	3.46
***T. castaneum* embedded**				3.41	1.46	3.84	1.68	0.89	2.58	1.05	1.18	1.75	1.48	0.923	1.55

Table S1. Relative dsDNA concentration (ng/µl) quantified with TapeStation for three specimens as independent counterparts at different times (T0, control; T1, one day; T2, three days; T3, seven days; T4, 30 days) from different insect orders (*Drosophila melanogaster*, Diptera; *Tribolium castaneum*, Coleoptera), under two different preservation scenarios (unembedded or embedded in pine resin). Only three data points are shown for T0 since at that time point control measurements were taken on specimens prior to starting the preservational treatments (*i.e.*, embedding them in resin or leaving them unembedded).

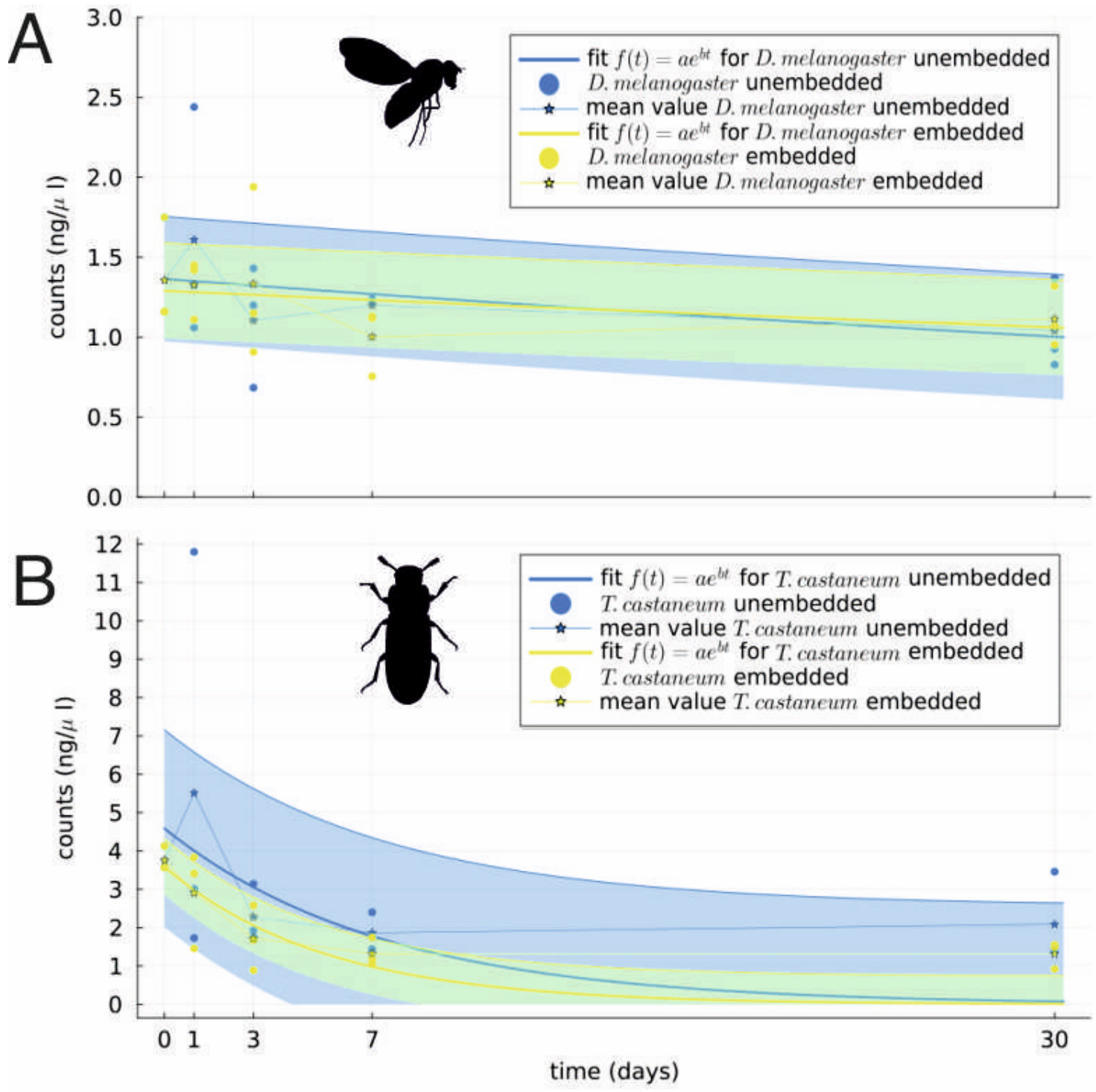

Figure S2. Overall trends of relative dsDNA concentration (ng/µl) over time obtained from independent individual time points based in the TapeStation quantification. **A**. Relative dsDNA concentration in *Drosophila melanogaster* specimens, both embedded in resin (yellow) and unembedded (blue). Solid lines correspond to the fit of the exponential function, and the shaded areas represent the region around the fitted values within the standard error. **B**. Relative dsDNA concentration in *Tribolium castaneum* specimens, under the same two conditions.

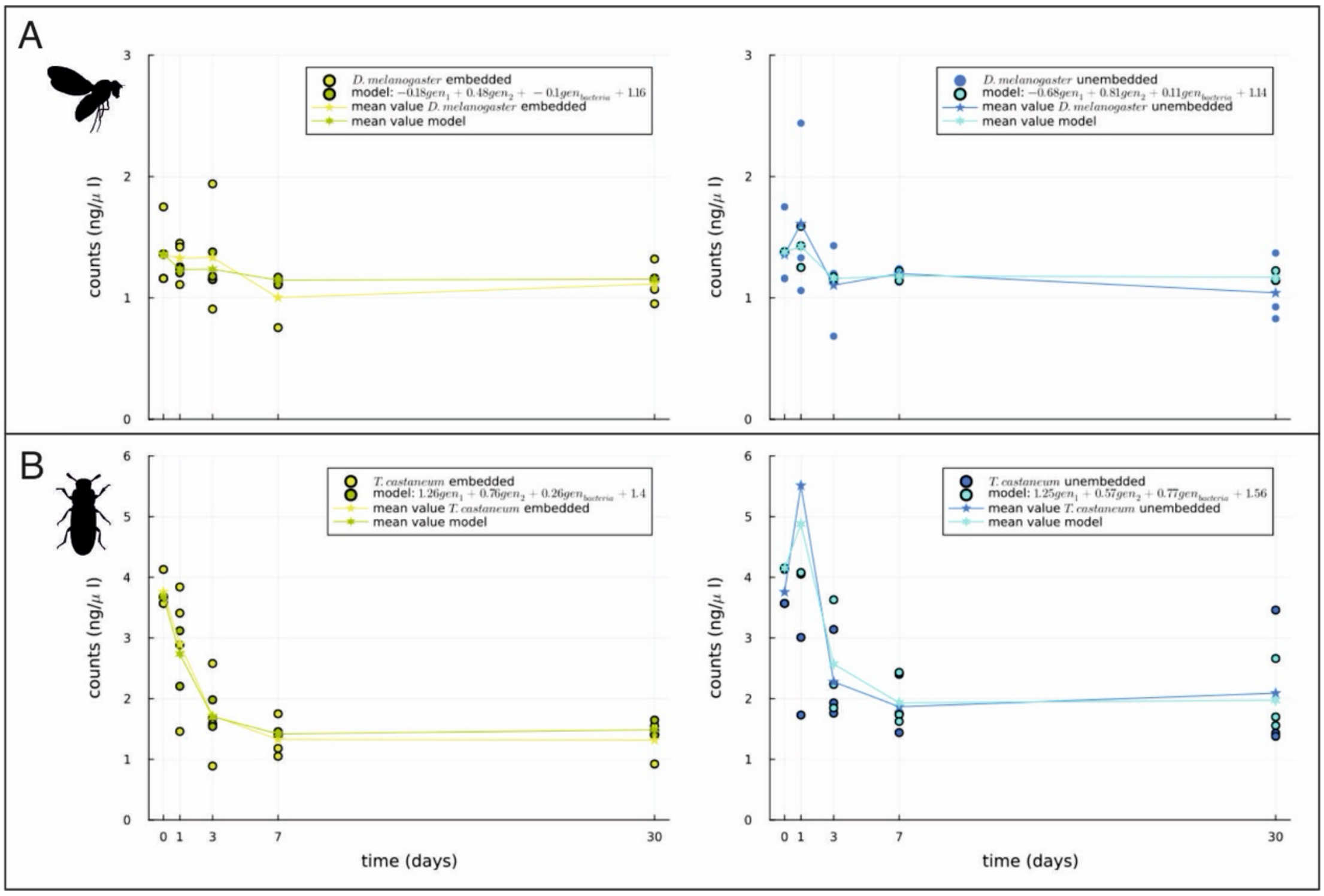

Figure S3. Linear Mixed Model Analysis representing the comparison between the genomic DNA obtained after TapeStation quantification and the mixed model. **A**. *Drosophila melanogaster* embedded and unembedded. **B**. *Tribolium castaneum* embedded and unembedded. It is important to note that the last value, β_0, is always close to 1. This is because 1 is the error limit of the machine.

In *D. melanogaster*

	Rpl32					*Hr3*					Bacteria				
	T0	T1	T2	T3	T4	T0	T1	T2	T3	T4	T0	T1	T2	T3	T4
Unembedded in resin	1	0.5046	0.0035	0.0006	0.0013	1	0.2822	0.0006	0.0005	0.0008	1	0.6605	0.0009	0.3395	0.0113
	1	0.2064	0.0005	0.0010	0.0015	1	0.1062	0.0004	0.0007	0.0009	1	0.1360	0.1839	0.0025	0.0300
	1	0.8695	0.0067	0.0154	0.0105	1	0.5804	0.0021	0.0037	0.0077	1	1.2672	0.2591	0.6358	0.7153
Embedded in resin	1	0.2532	0.0102	0.0006	0.0009	1	0.1150	0.0021	0.0004	0.0003	1	0.0673	0.1305	0.0101	0.0030
	1	0.1772	0.0518	0.0193	0.0007	1	0.0210	0.0020	0.0008	0.0004	1	0.0152	0.0855	0.0026	-
	1	0.1899	0.5390	0.0095	0.0082	1	0.0725	0.0196	0.0035	0.0028	1	0.3257	0.3540	0.5790	0.2564

In *T. castaneum*

	Rpl32					*Egfr*					Bacteria				
	T0	T1	T2	T3	T4	T0	T1	T2	T3	T4	T0	T1	T2	T3	T4
Unembedded in resin	1	0.7535	0.1733	0.0199	0.0083	1	0.8409	0.1285	0.0155	0.0090	1	1.3134	0.5409	0.1946	0.1642
	1	0.3614	0.0161	0.0272	0.0003	1	0.3802	0.1566	0.0306	0.0003	1	2.3839	0.1121	0.0193	0.0011
	1	1.2411	0.4703	0.0621	0.3552	1	1.1688	0.2088	0.0186	0.1416	1	3.6008	1.9977	1.0595	0.9384
Embedded in resin	1	0.6492	0.0576	0.0005	0.0000	1	0.6220	0.0497	0.0003	0.0001	1	0.8113	0.3205	0.0220	0.0730
	1	0.2393	0.0508	0.0004	0.0001	1	0.3143	0.0537	0.0001	0.0000	1	0.8878	0.1500	0.0024	0.0010
	1	0.9054	0.2646	0.0032	0.0578	1	0.2822	0.0767	0.0019	0.0984	1	2.6087	1.1006	0.2100	0.3161

Table S2. Individual qPCR measurements. DNA relative quantifications levels were assessed using comparative quantification and normalized against the genomic DNA amount in a reference sample taken at the start of the experiment. The measurement at T0 was defined as 1, and all later time points are shown as values relative to this baseline. Results are expressed as relative abundance in arbitrary units.

Mathematical Model of DNA degradation without replication

In the model we assume that the DNA under study is represented as a string of N characters. Within this string, the word X, consisting of n characters and corresponding to the genes of interest, appears repeatedly. At each time step, one character of the DNA string is selected randomly and is deleted with probability p. We then ask how the number of non-empty characters in the DNA string and the number of repetitions of X within the string will decay over time.

Here we assume that at each time step the choice of the character to be deleted may include characters that have already been deleted. That is, the DNA string always retains N positions, but some of them may be empty. We denote by $C(t)$ as the expected number of non-empty characters at time t, and by $X(t)$ as the number of times the word X is repeated at time t. Thus, the probability of selecting a non-empty character at time t is $C(t)/N$. Therefore, the expected deletion rate of characters is $pC(t)/N$, and the governing differential equation modelling this process is:

$$\frac{dC}{dt} = -p\frac{C(t)}{N},$$

whose solution is: $C(t) = Ne^{-pt/N}$.

A word X will be destroyed if any of its characters is deleted. This occurs if an occupied character is chosen (with probability $C(t)/N$) and that character belongs to one of the n positions of the word X (with probability $nX(t)/C(t)$). Thus, the destruction rate of words X is given by $-\frac{pnX(t)}{N}$, and the differential equation governing the behavior of $X(t)$ is:

$$\frac{dX}{dt} = -\frac{pnX(t)}{N},$$

whose solution is: $X(t) = X(0)e^{-pnt/N}$.

Both models coincide in being of the form ae^{-bt} (Saito et al., 2021), which is the model we fitted to the data.

Bacterial DNA model

This model does not account for the fact that there are DNA strands that can be replicated, as occurs in bacterial reproduction. Bacteria, in order to reproduce, depend on a substrate that we will call $S(t)$, which they will consume. Thus, $C(t)$ will only degrade in dead bacteria, but living bacteria will contribute to increasing the amount of genetic material. We will call $C_l(t)$ the portion corresponding to living bacteria and $C_d(t)$ the portion corresponding to dead bacteria. A reasonable model for $C_l(t)$, $C_d(t)$, and S is similar to the Lotka-Volterra predator-prey model (Lotka, 1925; Volterra, 1926):

$$\frac{dC_l}{dt} = \alpha S C_l - \beta C_l$$

$$\frac{dC_d}{dt} = \beta C_l$$

$$\frac{dS}{dt} = -\gamma C_l S$$

That is, the bacterial reproduction rate is proportional to the number of bacteria present and the amount of substrate present, minus the number of bacteria that die. The mortality rate is proportional to the number of living bacteria, and the substrate loss rate is proportional to the number of living bacteria. α, β, and γ are parameters related to the efficiency of converting food into bacterial births, the mortality rate, and the substrate consumption rate per bacterium, respectively.

Finally, to this set of equations, we can add the equation we already had:

$$\frac{dC}{dt} = -p\frac{C(t)}{N},$$

where $C(t) = C_l(t) + C_d(t)$.

Therefore, a minimal model considering bacterial reproduction includes seven parameters: $C_l(0), C_d(0), S(0), p, \alpha, \beta$ and γ. Considering that we had only data for five different times, we cannot fit this kind of model with our data robustly.

Fitted parameters

When adjusting the exponential model parameters, it is observed that the coefficients a for total DNA measurements are consistent. For embedded and unembedded *D. melanogaster*, these coefficients are 4.03 and 4.19, respectively, while for embedded and unembedded *T. castaneum*, they are 22.50 and 24.80, respectively (Figure S4 top panel). For relative DNA measurements, parameters remain consistent around 1(Figure S5 top panel), which is in keeping with the fact that the data are normalized with respect to the measurement at time 0.

Regarding the value of parameter b, the total DNA count yields 0.26, 0.97, 0.39, and 0.28 for embedded and unembedded *D. melanogaster* and embedded and unembedded *T. castaneum*, respectively (Figure S4 bottom panel). The case of unembedded *D. melanogaster* stands out, with a parameter value much higher than the rest. A similar pattern is observed for specific gene groups (Figure S5 bottom panel), where most values range between 0.4 and 1.4, except for resin-embedded *D. melanogaster*, where the parameter b is 2.66.

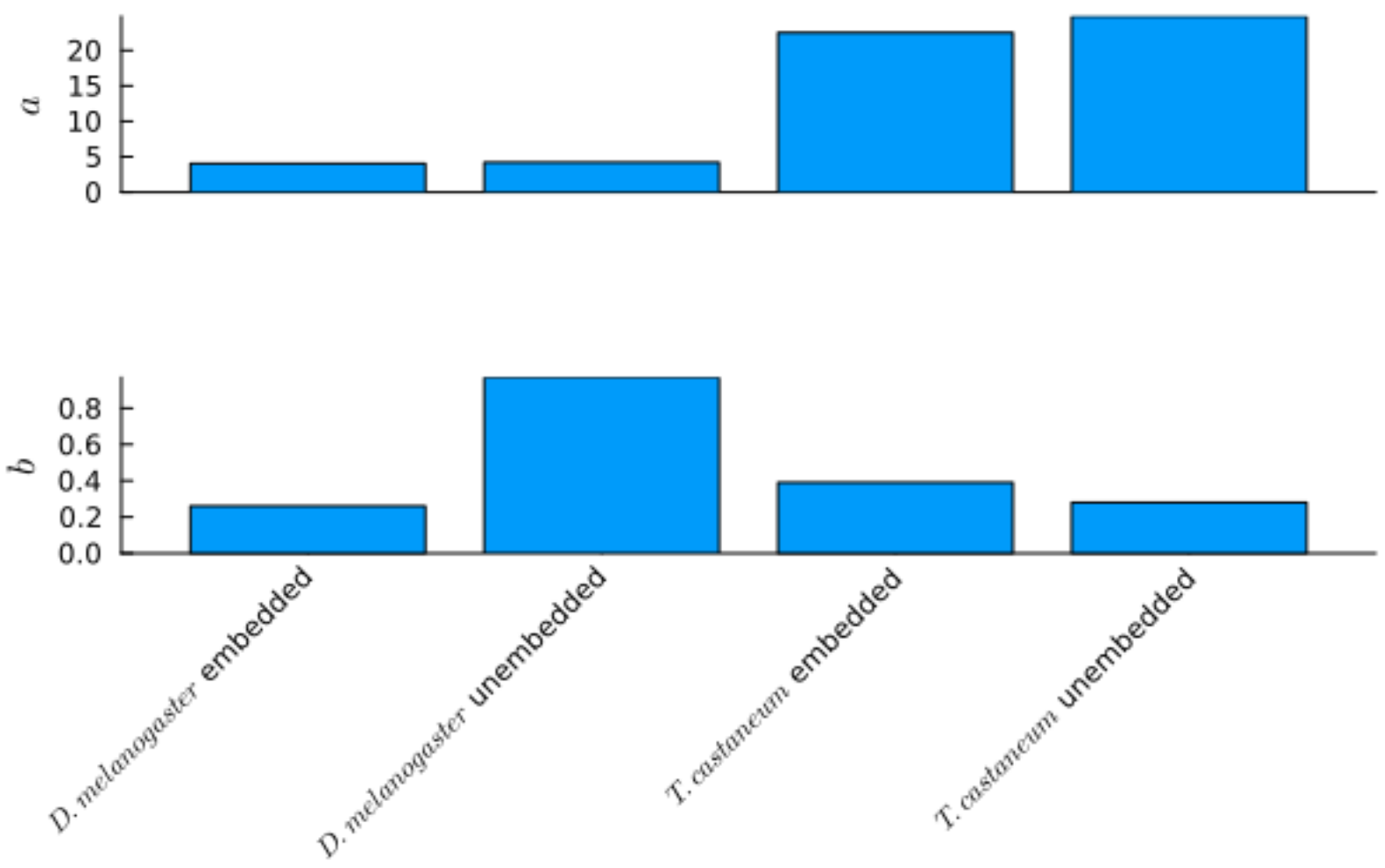

Figure S4. Parameters a and b of the exponential model ae^{-bt} for the genomic DNA concentration (ng/µl) over time obtained from independent individual time points based in the NanoDrop quantification. Parameter a is plotted in the top panel, whereas parameter b is plotted in the bottom panel.

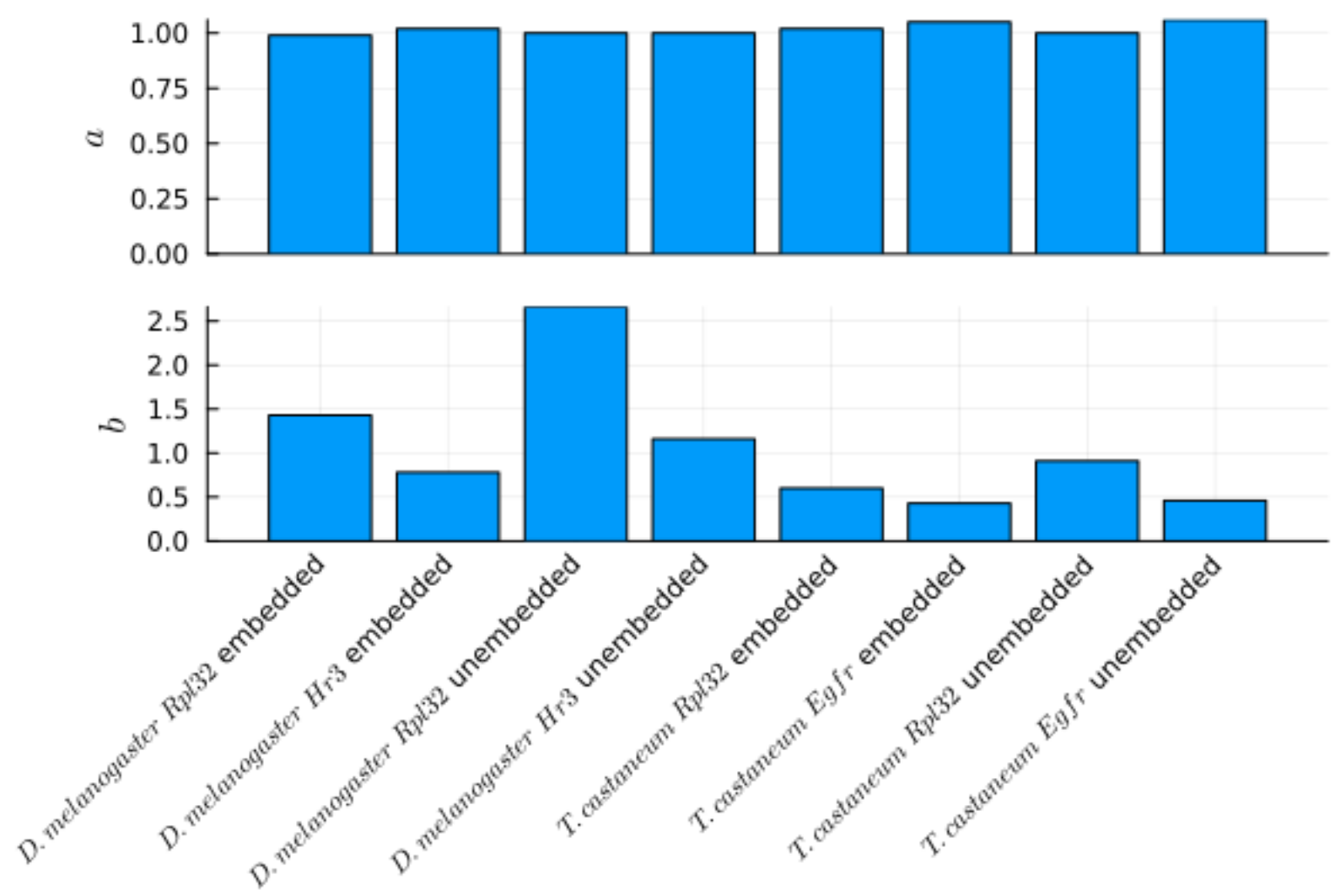

Figure S5. Parameters a and b of the exponential model ae^{-bt} for DNA relative quantifications using qPCR measurements. Parameter a is plotted in the top panel, whereas parameter b is plotted in the bottom panel.

References

Lotka, A. J. *Elements of Physical Biology* (Williams & Wilkins, 1925).

Saito, T. & Doi, H. A model and simulation of the influence of temperature and amplicon length on environmental DNA degradation rates: A meta-analysis approach. *Front. Ecol. Evol.* **9**, 623831 (2021).

Volterra, V. Variazioni e fluttuazioni del numero d'individui in specie animali conviventi. *Memorie della Reale Accademia Nazionale dei Lincei* **2**, 31–113 (1926).
